# Automatic segmentation of multi-echo Cardiac Magnetic Resonance images

**DOI:** 10.1186/1532-429X-11-S1-P263

**Published:** 2009-01-28

**Authors:** Gilion Hautvast, Yansong Zhao, Marc Kouwenhoven, Marcel Breeuwer

**Affiliations:** 1 Philips Healthcare, Best, Netherlands; 2 Philips Healthcare, Cleveland, OH USA

**Keywords:** Cardiac Magnetic Resonance, Iron Overload, Iron Chelation Therapy, Exponential Decay Curve, Automatic Delineation

## Introduction

Patients with thalassaemia major develop iron overloading due to regularly required blood transfusions, which is treated with iron chelation therapy. To monitor the effect of this treatment the hepatic and cardiac myocardial iron content is monitored on a regular basis. Clinical management guidelines for these patients include a yearly Cardiac Magnetic Resonance (CMR) exam [1], containing a multi-echo scan to enable T2* quantification for iron load assessment. The introduction of T2* CMR, alongside other improvements in clinical care, has improved survival in patients with thalassaemia major significantly [2].

Quantification of the myocardial T2* from multi-echo CMR images can be performed by fitting an exponential decay curve to the signal obtained by averaging image intensities within a region of interest in the myocardial septum in all echos [3].

## Purpose

The purpose of our work is to simplify the analysis of multi-echo CMR images by developing and technically validating an automatic delineation method for the left ventricular contours in multi-echo CMR images.

## Methods

The starting point for our new method was our earlier-developed method for cine CMR images [4]. Similarly to that method, our new method starts by locating the LV contours by detecting ring structures, followed by a greedy optimization of a deformable template [5] in a coarse-to-fine approach to obtain accurate myocardial contours. The new method assumes a bright myocardium and a dark blood pool. Furthermore, contour detection is performed at the first echo only, after which the contours are copied to all other echos.

We have used 28 black-blood multi-echo CMR scans from 10 patients. Each scan consisted of 4–8 echos at TE 1.3–17 ms and the isotropic image resolution was 0.94–1.45 mm. We are grateful to Children's Hospital Boston and Bioiatriki, Athens for providing us with clinical image data. Golden standard contours were defined manually on all images.

The technical validation of our method includes an a assessment of the contour quality and the effect on the T2* value. Contour quality was assessed by measuring Root-Mean-Square (RMS) contour positioning errors between the golden standard and detected contours. The effect on the T2* value was assessed by calculating the mean absolute difference and correlation coefficient between T2* values obtained from the automatically detected contours and the golden standard contours.

To fit the exponential decay curve we have used the Ratio-Least-Squares algorithm [6], in which a weighted fit is computed using decreasing weights for later echos. Especially in patients with severe iron overload, this is important as the image intensity may decrease below noise levels due to a short T2*.

## Results

**Figure 1:**
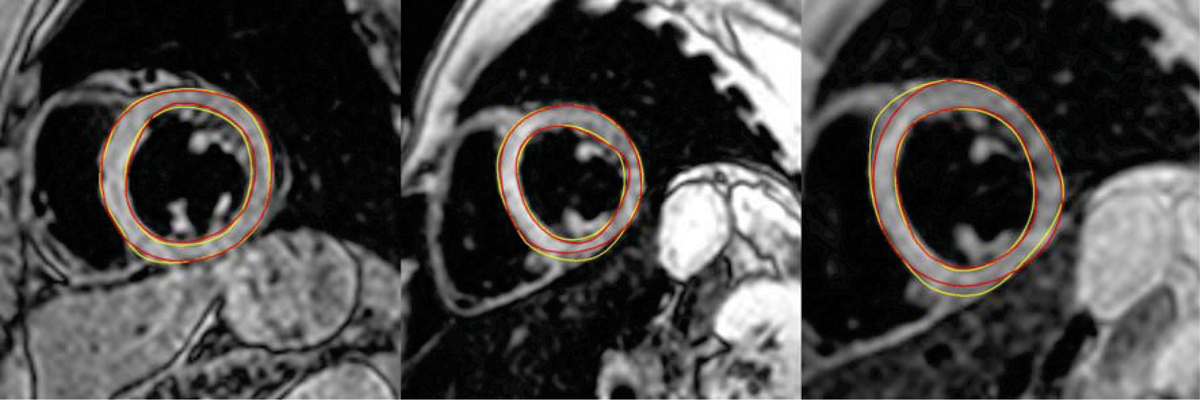
**Examples of resulting LV contours (red) and the golden standard (yellow)**.

**Figure 2:**
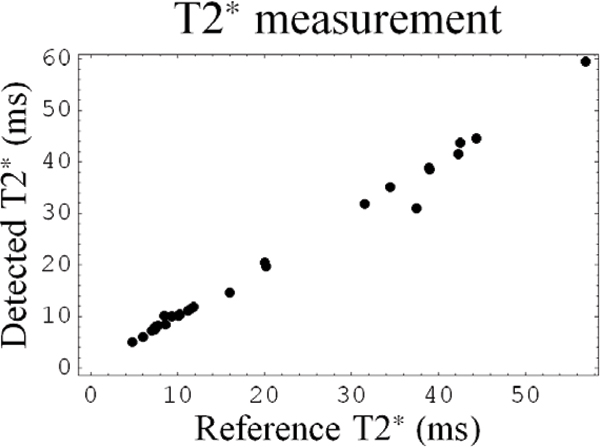
2T2* from detected contours versus T2* from manual contours.

All images were delineated successfully (Figure 1). We found RMS contour positioning errors of 1.42 ± 0.61 mm for the LV endocardium, and 1.36 ± 0.96 mm for the LV epicardium. T2* values derived using automatically obtained contours differed on average 0.68 ms from T2* values derived from manually defined contours and correlated very well (r^2^ = 0.99) (Figure 2). Furthermore, the average deviation in T2* in iron overloaded patients (T2* < 20 ms) was even lower (0.36 ms).

## Conclusion

We have developed and technically validated an automatic delineation algorithm for multi-echo CMR images. The resulting contours are accurate and allow for accurate T2* quantification.
